# Plants as biofactories for production of the aphid sex pheromone nepetalactone

**DOI:** 10.1111/ppl.70110

**Published:** 2025-02-16

**Authors:** Abraham Ontiveros‐Cisneros, Jule Salfeld, Bao‐Jian Ding, Hong‐Lei Wang, Oliver Moss, Magne Friberg, Alex Van Moerkercke, Christer Löfstedt, Olivier Van Aken

**Affiliations:** ^1^ Department of Biology Lund University Lund Sweden; ^2^ Present address: Current address: Faculty of Biology University of Freiburg Freiburg Germany; ^3^ Present address: Current address: Plant Breeding Department Swedish University of Agricultural Sciences Alnarp Sweden

## Abstract

Aphids cause massive agricultural losses through direct damage or as pathogen vectors. Control often relies on insecticides, which are expensive and not selective. An interesting alternative is to use aphid sex pheromones nepetalactone (NON) and nepetalactol (NOL) to interfere with aphid mating or attract aphid predators. Here, we explore production of these compounds in plants, as their precursors can be derived from mevalonate (MVA) and methylerythritol phosphate (MEP) pathways. By introducing six genes, including a major latex protein‐like (MLPL) enzyme, we engineered a functional nepetalactol biosynthetic pathway into plants. Transient expression of these enzymes in *N. benthamiana* caused production of nepetalactone, without the need for external supplementation with substrates. Targeting all six enzymes into the chloroplast appeared to result in higher NON yields than just chloroplast‐targeting the first two enzymes. We could not detect NOL, suggesting it is rapidly oxidised to NON. In addition, we produced NON in stably transformed *Camelina sativa* (Camelina) lines. Interestingly, the specific NON enantiomer was different in *N. benthamiana* compared to in Camelina, indicating the value of different platforms for producing specific isoforms. This opens possibilities for using plants as green factories of pheromones for baits or as pheromone dispensers that interfere with insect behaviour.

## 1 INTRODUCTION

Aphids (Hemiptera, Aphidoidea) are well represented with thousands of species recorded, of which around 100 species are detrimental to the agricultural landscape. Most of these crop pests belong to the Aphidinae subfamily, which feeds on herbaceous plants by sap‐sucking (Blackman and Eastop, 2017). In temperate climate conditions, aphids can colonize up to 25% of all plant species. With climate change, it is expected that large, previously inhospitable areas will become suitable for aphid invasion and reproduction (Dedryver et al., 2010; Pritchard and Vickers, 2017). Worldwide, aphids are already responsible for the loss of up to billions of USD in crop yield (Loxdale et al., 2017). The ability to cause this damage is due to aphids' high reproductive potential, adaptability, and dispersal capacities. For example, they can vary their morphology, build chemical defences, facultatively produce winged and wing‐less morphs, and form symbiotic associations (Dedryver et al., 2010; Loxdale et al., 2017). Also, they can reproduce sexually and asexually, which translates into both having the ability to rapidly increase in numbers through clonal development and to adapt through gamete recombination (Sorensen, 2009; Simon and Peccoud, 2018).

Aphids cause crop damage in several different ways. They feed from plant phloem sap, causing withering and death, facilitate sooty mold growth caused by aphid excrement, and have phytotoxic saliva that carries viruses and fungi (Stevens and Lacomme, 2017; Sorensen, 2009). Many of these viruses have major economic impact, such as *Barley yellow dwarf viruses, Cereal yellow dwarf viruses, Beet yellows virus, Beet mild yellowing virus, Cucumber mosaic virus, Potato virus Y* and *Potato leaf roll virus* (Dedryver et al., 2010). Therefore, considerable efforts are made to generate efficient aphid pest control measures.

Historically, aphid control has relied on chemicals such as nicotine sulphate, dichloro‐diphenyl‐trichloroethane (DDT), organochlorinated compounds, organophosphorus, carbamates and pyrethroids. Typically, these are expensive compared to the yield loss they prevent and/or not particularly targeted at aphids. For example, neonicotinoids constitute one of the most effective treatments, but they are similarly efficient at killing beneficial insects, such as natural enemies and pollinators (Dedryver et al., 2010; Devonshire, 1989; Van der Sluijs et al., 2013). Therefore, efforts have been made to breed aphid resistance into plant materials, which is more environmentally sustainable and can be obtained through traditional approaches. However, the disadvantages are that such resistance traits can interfere with yield, and native sources of resistance could be limited or non‐existent. Furthermore, if resistance is monogenic, aphids can overcome it through evolving resistance (Van der Arendt et al., 1999), just like they are ultimately destined to adapt to synthetic pesticides (Simon and Peccoud, 2018).

Biological control represents an alternative for managing aphid pest populations and relies on the use of aphid natural enemies. These can be microorganisms like fungi (*Entomophthorales* and *Hyphomycete*), predators like lacewings, aphid midges, ladybird beetles, and parasitoid‐like parasitic wasps. For example, the parasitoid wasp *Aphelinus mali* is used to control the woolly apple aphid in France and the parasitoid *Aphidius rhopalosiphi* was effective, but not as economically profitable, in controlling cereal aphids (Blommers, 1994; Levie et al., 2005).

One promising and sustainable approach to aphid pest control is the use of aphid pheromones. In 1973, it was discovered that aphids release an alarm pheromone, β‐farnesene, when disturbed by predators, which causes dispersal behavior in surrounding individuals (Nault et al., 1973). In previous studies, this particular pheromone was proven to reduce aphid colonization in transgenic *A. thaliana* expressing β‐farnesene synthase from *Mentha piperita* (Beale et al., 2006). However, it was later proven that plants emitting β‐farnesene continuously did not serve as an effective defense measure, as aphids got habituated to the presence of the volatile (Kunert et al., 2010). As mentioned before, aphids can reproduce sexually. For this to happen, females release a sex pheromone mix to attract conspecific males, consisting mainly of the iridoids (4aSR, 7SR, 7aRS)‐nepetalactone and (1RS, 4aSR, 7SR, 7aRS)‐nepetalactol, where nepetalactol (NOL) is oxidized to the ketone nepetalactone (NON) (Dawson et al., 1987; Dewhirst et al., 2010; Dawson et al., 1990). The ratio of these compounds respective to each other seems to be determinant for an increased response of males towards their conspecific female (Dewhirst et al., 2010; Hardie et al., 1990). Sex pheromones could be used as a form of pest control by releasing these compounds to cause mating disruption. Also, they can be dispensed by man‐made baits for mass trapping or to attract aphid predators. There have been studies proving the attraction of aphid predators towards pheromone‐releasing devices, showing larger aphid predator populations on plants closer to these lures (Glinwood et al., 1998; Boo et al., 1998).

**FIGURE 1:**
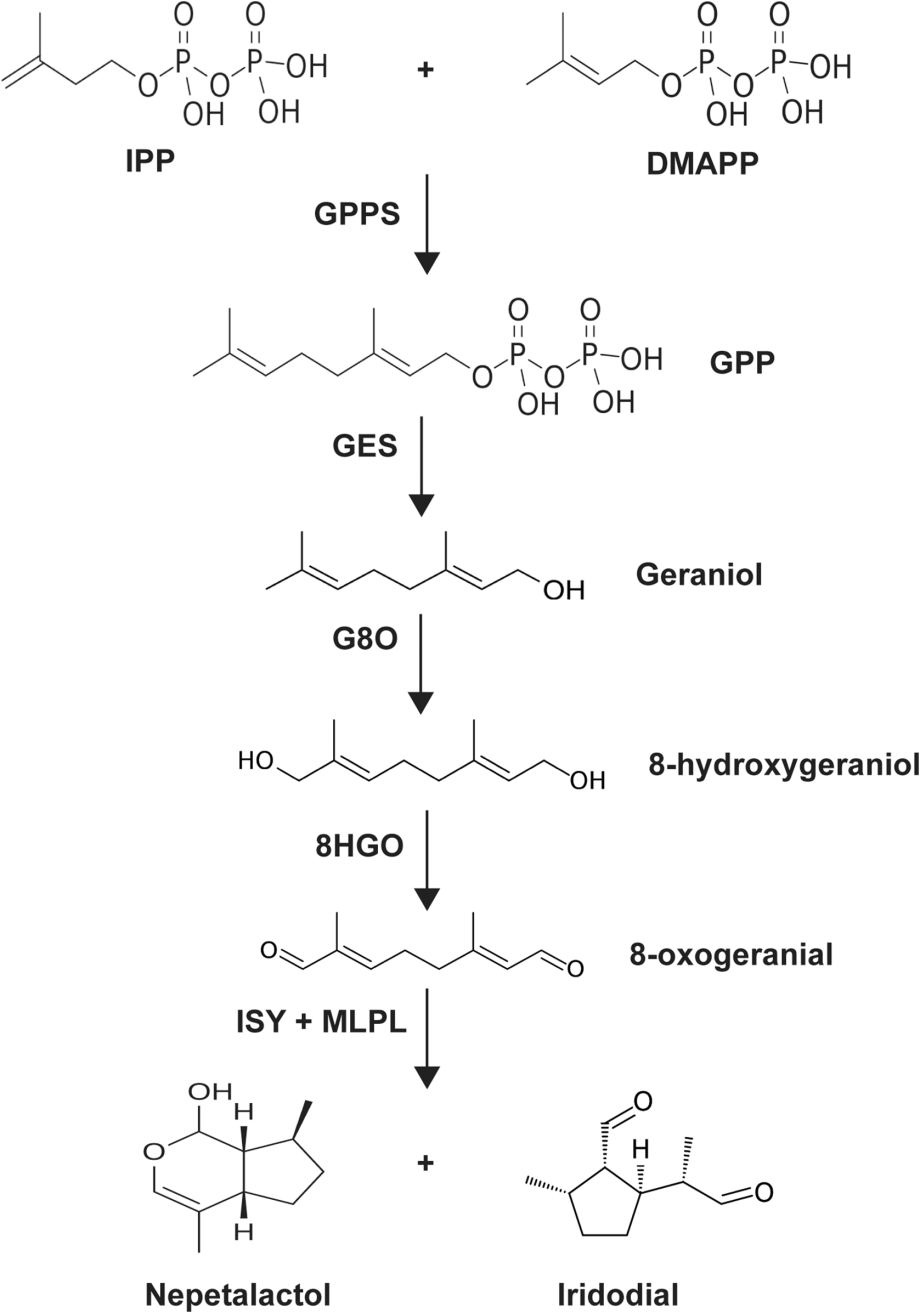
NOL biosynthetic pathway. All enzymes are based on *C. roseus* seco‐iridoid pathway with the addition of MLPL from *Nepeta mussinii*. IPP = isopentenyl pyrophosphate, DMAPP = dimethylallyl pyrophosphate, GPP = geranyl diphosphate, GPPS = GPP synthase, GES = geraniol synthase, G8O = geraniol 10 hydroxylase/8‐oxidase, 8HGO = 8‐hydroxygeraniol oxidoreductase, ISY = iridoid synthase, MLPL = major latex protein‐like protein.

Production of NON and NOL is not exclusive to insects, but also occurs in plants. For example, species of the genus *Nepeta* produce NON, while *Catharanthus roseus* is capable of NOL production through the seco‐iridoid pathway (Formisano et al., 2011; Miettinen et al., 2014). Terpene production in both plants and insects starts with isopentenyl diphosphate (IPP) and dimethylallyl diphosphate (DMAPP), which are derived from the cytosolic mevalonate pathway (MVA) or the plastidial methylerythritol phosphate pathway (MEP) (Beran et al., 2019; Yang et al., 2012). The first step in NOL biosynthesis (Figure 1) is the condensation of IPP and DMAPP into geranyl diphosphate (GPP) by an isoprenyl diphosphate synthase (IDS): GPP synthase (GPPS) in *C. roseus* and ApIDS in the aphid *Acyrthosiphon pisum* (Miettinen et al., 2014; Köllner et al., 2022). GPP is then hydrolyzed to form geraniol by geraniol synthase (GES) in *C. roseus* and by ApGES in *A. pisum* (Köllner et al., 2022; Simkin et al., 2013). Geraniol is then hydroxylated to 8‐hydroxygeraniol by the cytochrome P450 monooxygenase geraniol 10‐hydroxylase/8‐oxidase (G8O) in *C. roseus* and geraniol 8‐hydroxylase (ApG8H) and a P450 reductase (ApRed) in *A. pisum* (Köllner et al., 2022; Collu et al., 2001). In the next step, 8‐hydroxygeraniol is oxidized to 8‐oxogeranial by 8‐hydroxygeraniol oxidoreductase (8HGO) in *C. roseus* and the putative farnesol dehydrogenase ApHGO in *A. pisum* (Miettinen et al., 2014; Köllner et al., 2022). The product 8‐oxogeranial is then cyclized to *cis‐trans*‐iridodial and *cis‐trans*‐NOL. In plants, this step is performed by the enzyme iridoid synthase (ISY), assisted by major latex protein‐like protein (MLPL) and nepetalactol‐related short‐chain dehydrogenase (NEPS) enzymes in *Nepeta* (Geu‐Flores et al., 2012; Lichman et al., 2020; Lichman et al., 2019; Dudley et al., 2022). As for aphids, the polyprenol reductase ApISY is responsible for this step (Köllner et al., 2022). NOL can then be converted into *cis‐trans*‐NON by NEPS protein in *Nepeta* and by the nepetalactol oxidase ApNEPO in *A. pisum* (Köllner et al., 2022; Lichman et al., 2019).

As NOL is derived from IPP and DMAPP, precursors that are part of the plant MVA and MEP pathways, the aim of this study was to engineer the listed metabolic steps into plant systems to produce NOL/NON sex pheromone components. There has been a previous study where this pathway, as part of the secaloganin biosynthesis pathway, has been transiently reconstituted in *N. benthamiana*, producing final products, but only when externally supplementing intermediates (Miettinen et al., 2014). Complementing this, Dudley et al. (2022) managed to produce strictosidine (a product further downstream in the pathway) by adding MLPL enzyme and further downstream steps. They obtained the highest yield when targeting the first two steps (GPPS and GES) to the chloroplast, rather than by targeting all enzymes to the chloroplast. This result could be explained by farnesyl diphosphate synthase (FPPS) competing for cytosolic GPP to produce farnesyl diphosphate (FPP) (Dudley et al., 2022).

Engineering plants capable of producing aphid sex pheromones provides the possibility of using them as green biofactories. In the literature, there are several examples of crop plants, e.g. Camelina, being used as platforms for the efficient production of insect pheromones (Wang et al., 2022; Xia et al., 2021). The extracted compounds could be used in traps, or pheromone‐producing plants could be used as natural dispensers to induce mating disruption or attract aphid predators (Löfstedt and Xia, 2021).

For example, the coccinellid beetle *Coleomegilla maculata* has been shown to be responsive to NOL and NON in an olfactory assay, while lacewings *Chrysoperla carnea* and *Chrysopa cognata* are reactive to NOL and NON, and *Chrysopa oculata* to synthetic NOL (Zhu et al., 1999; Boo et al., 1998; Zhu et al., 2005).

In the first part of this study, we used different cloning techniques to build a seco‐iridoid metabolic pathway using plant‐derived enzymes, while later we added RecA chloroplast targeting sequences (Cerutti et al., 1992) plus a MLPL enzyme to our metabolic engineering strategy. The generated constructs were used for transient transformation of *N. benthamiana* leaves and stable transformation of Camelina as a potential crop to be implemented as a biofactory. Transformed and infiltrated plants were evaluated at the gene expression level and for NOL/NON pheromone production via GC/MS analyses.

## 2 MATERIALS AND METHODS

### 2.1 DNA assembly and cloning

The gene sequences corresponding to NOL pathway have the following GenBank accessions: *GPPS* from *Picea abies* (EU432047.2), and *GES* (KF561459.1), *G8O* (KF561461.1), *8HGO* (KF302069.1), and *ISY* (KJ873886.1) from *Catharanthus roseus*. Plasmids containing these gene sequences were kindly provided by Johan Memelink (Leiden University). These plasmids were then used as templates to create two polycistronic constructs: GES‐GPPS and G8O‐8HGO‐ISY. For the GES‐GPPS construct, a T2A sequence (Szymczak‐Workman et al., 2012) was added in between both gene sequences. In the case of G8O‐8HGO‐ISY construct, an E2A sequence was added between *G8O* and *8HGO* and a P2A sequence between *8HGO* and *ISY*. These added sequences belong to a group of “self‐cleaving” 2A peptides. These originally are viral peptides that form a secondary structure and cause ribosome skipping (Liu et al., 2017). This skipping allows an mRNA sequence to be translated as two separate peptides.

Gene sequences were amplified via PCR using Phusion Hot Start Flex DNA Polymerase with primers designed to remove stop codons while adding the mentioned 2A sequences as well as attB1/2 attachment sites for subsequent Gateway cloning (Table S1; Figure S1). Gateway vectors used were pDONR221 as a donor and pH2GW7 (hygromycin resistance) for the GES‐GPPS construct and pB2GW7 (Basta resistance) for the G8O‐8HGO‐ISY construct as destination vectors (Karimi et al., 2002). Sanger sequencing was used to confirm correct construct sequence. Resulting vectors were transformed via heat‐shock method and propagated in *E. coli* DH5α in LB plates supplemented with 50 mg mL^−1^ kanamycin for pDONR221 or 50 mg mL^−1^ spectinomycin for pB2GW7 and pH2GW7, respectively.

Further destination vectors were generated to contain all required metabolic steps for NOL biosynthesis, including MLPL, as individual expression cassettes, with and without chloroplast targeting of the enzymes (Figure 3). The second round of cloning (Figure 3) was done with the purpose of creating a construct containing all the metabolic steps for NOL biosynthesis along with an herbicide resistance gene and without using the 2A peptide sequences. Also, according to a recent study, biosynthesis of iridoids is boosted when chloroplast targeting is used, especially for GES and GPPS, and when the MLPL enzyme is included in the metabolic pathway (Dudley et al., 2022). Therefore, we decided to create two versions of this construct with MLPL: one with all genes having a RecA chloroplast targeting sequence on the 5′ end, and one with only *GES* and *GPPS* containing the RecA sequence. For this purpose, GES‐GPPS and G8O‐8HGO‐ISY constructs were used as templates for the amplification of the genes *GPPS*, *GES*, *G8O*, *8HGO* and *ISY*. Apart from these, the *MLPL* gene was amplified from *Nepeta mussinii* cDNA and its sequence corresponded to the GenBank accession (MT108267.1). Along with the genes related to NOL biosynthesis, a Basta resistance gene expression cassette driven by a CaMV 35S promoter and a nopaline synthase (NOS) terminator was also synthesized using ThermoFisher GeneArt Gene synthesis service.

As each gene requires to be transcribed individually, a CaMV 35S promoter and different terminators were added, flanking each gene sequence at the 5′ and 3′ ends, respectively. For N‐terminal chloroplast targeting of the enzymes, a RecA sequence was added between the promoter and the gene sequence (Table S2). Cloning of these individual expression cassettes was performed using a Gibson Assembly Master Mix (New England BioLabs Inc). The assembly of the 7‐gene constructs (GPPS‐GES‐G8O‐8HGO‐ISY‐MLPL‐BASTA) was done in two steps. First, gene pairs were formed using NEBridge Golden Gate Assembly Kit (New England BioLabs Inc) following the manufacturer's specifications and NEB Builder website for suitable primer design (Table S3). The gene pairs *GPPS*‐*GES*, *G8O*‐*8HGO* and *ISY*‐*MLPL* were then amplified using specific attB primers for further Multisite Gateway cloning (Table S4) (Petersen and Stowers, 2011). Each gene pair, as well as the Basta resistance gene, was cloned into a specific version of pDONR221 during a BP reaction with 100 ng of the PCR product,100 ng of the corresponding pDONR221 and 0.6 μL of BP clonase II enzyme mix from Invitrogen. The reaction was performed overnight at room temperature. All resulting vectors were transformed via heat‐shock method and propagated in *E. coli* DH5α in LB plates with 50 mg mL^−1^ kanamycin. Colonies were evaluated using colony PCR and the construct's sequence was confirmed via Sanger sequencing. Purified plasmids were obtained from these colonies and used in an LR reaction with pXZ393b as destination vector. For each entry vector, 100 ng were required, along with 150 ng of destination vector and 1 μL of LR clonase II Plus enzyme from Invitrogen. This reaction was incubated at room temperature for 48 h. After the first 24 h, a second addition of 1 μL of LR clonase was performed. The resulting plasmids were transformed following Proteinase K treatment into Promega *E.coli* HB101 cells according to the manufacturer's protocol. Colonies were grown in LB plates with 50 mg mL^−1^ spectinomycin. Colonies were evaluated using colony PCR and the construct's sequence was confirmed using whole plasmid sequencing (Plasmidsaurus Inc.).

For both cloning approaches, sequenced plasmids were introduced into chemically competent *Agrobacterium tumefaciens* strain GV3101 by heat‐shock transformation. Selection of transformed bacteria was done on LB plates containing 100 mg mL^−1^ spectinomycin, 100 mg mL^−1^ rifampicin and 40 mg mL^−1^ gentamycin.

### 2.2 Stable transformation and selection in *C. sativa*

In this study, *C. sativa* cv. Suneson was used for stable transgenic plant line generation via floral dipping using *A. tumefaciens* carrying the appropriate vectors as described in previous literature (Lu and Kang, 2008). For this purpose, Camelina plants were grown as previously described in literature (Ontiveros‐Cisneros et al., 2022). From pB2GW7 and pXZ393b‐transformed plants, seeds were collected, densely grown on soil trays, left for 2 days of stratification at 4°C and sprayed with 100 mg L^−1^ Basta as reported in literature (Nguyen et al., 2013; Ontiveros‐Cisneros et al., 2022). Basta selection was performed by spraying when plants reached the two‐leaf stage (approx. one week old), and later by two more rounds of spraying approx. every three days. Survivors were used for further analyses. For T2 onwards, 50 seeds per line were gas sterilized by using a chlorine gas sterilization method with a solution of 40 mL MQ water, 6.25 mL bleach and 1.75 mL HCl and left overnight inside a desiccator. Seeds were then plated and counted on MS media at pH 5.7 with 15 mg L^−1^ Basta to obtain homozygous lines. As for pH2GW7‐transformed plants, selection was done using MS media plates at pH 5.7 with 22.5 mg L^−1^ hygromycin with gas‐sterilized seeds as previously described (Ontiveros‐Cisneros et al., 2022).

### 2.3 Camelina crosses

To generate plants containing the five essential metabolic steps for NOL production from 2A peptide vector‐transformed plants, we crossed GPPS‐GES with G8O‐8HGO‐ISY transformed plants (Ontiveros‐Cisneros et al., 2022). Crosses using GPPS‐GES (pH2GW7) flowers and G8O‐8HGO‐ISY (pB2GW7) pollen were performed as described in our previous study (Ontiveros‐Cisneros et al., 2022). Collected seeds from resulting siliques were grown on soil and sprayed with Basta to select for the presence of the paternal construct. Further generations were sown in MS media plates containing the appropriate amounts of both hygromycin and Basta.

### 2.4 Transient expression in *N. benthamiana*

*Nicotiana benthamiana* (WT) plants were used as a fast way to evaluate the expression of desired transgenes via transient transformation, infiltrating the underside of the leaves with *A. tumefaciens* carrying the desired vector. This procedure was performed as described by Wood et al. (2009) with 6‐week‐old plants grown under long day conditions (16 h light/8 h dark, approximately 400 μmol photons m^−2^ s^−1^) using Aura Light T8 36 W/840 lamps. Along with the desired vectors, a strain of *A. tumefaciens* carrying a *P19* gene was co‐infiltrated in every leaf, as it prevents post‐transcriptional gene silencing (Garabagi et al., 2012).

### 2.5 Gene expression analysis

RNA was extracted from leaf samples (~30 mg) from plants surviving antibiotic selection with Spectrum™ Plant Total RNA Kit (Sigma). RNA samples were normalized to 100 ng mL ^−1^ and cDNA was synthetized from 500 ng of RNA per sample using Bio‐Rad iScript Reverse Transcription Kit. Quantitative Real Time PCR (qRT‐PCR) program was as follows: 5 min at 25°C, 25 min at 42°C and 1 min at 95°C. Samples were diluted 7:1 with MQ water. For qRT‐PCR, primer pairs were designed to amplify ~120 bp products (Table S1). For *C. sativa*, *ACTIN2* (ACT2; XM_010489499.1) was used as housekeeping gene for relative expression analysis. The qRT‐PCR mix was made with 2.5 μL Sso Advanced™ Universal SYBR Green Supermix, 0.25 μL primers (10 mM) and 0.25 μL H_2_O, along with 2 μL of diluted cDNA. The qRT‐PCR program was performed in a BIO‐RAD CFX384 Touch Real‐Time System following previously described methods (Broda and Van Aken, 2018). For all relative gene expression analyses, the obtained values were normalized considering untransformed Camelina or *N. benthamiana* as the reference value of expression.

### 2.6 Volatiles analysis‐ Solvent extraction and GC/MS

Leaf samples (50 mg) from Camelina crosses were frozen in 2 mL microcentrifuge tubes containing 2 metal beads and ground to fine powder using an MM300 tissue lyser (Retsch) twice for 30 s at a frequency of 24 s^−1^ in pre‐cooled cassettes. Grinding of 500–750 mg transiently transformed *N. benthamiana* leaf tissue was performed in liquid nitrogen using a mortar and pestle. Powder was transferred to glass vials, where 1 mL of dichloromethane (DCM) was used as a solvent to extract the volatiles from the plant tissue for 2 h. Extracts were transferred to empty glass vials and concentrated to 200 μL using a constant N_2_ flow at 1.5–2 bar. Further on, a column of 100 mg Florisil® (MgO_3_Si) (Sigma Aldrich) was used to filter the extract, which was concentrated down to 200 μL before injecting to the GC–MS. Volatiles were analysed using an Agilent Technologies 7890A GC system coupled to an Agilent Technologies 5975C mass selective detector. The GC system was equipped with non‐polar HP‐5MS Ultra Inert column (30 m x 0.25 mm, 0.25 μm film thickness) and was operated using helium as a carrier gas (1 mL min^−1^ flow). The method started with the oven temperature set at 60°C for 1 min, then a temperature ramp of 10°C min^−1^ until reaching 230°C, a second ramp of 20°C min^−1^ until reaching 250°C and holding it for 5 min.

### 2.7 Volatiles analysis‐ SPME and GC/MS

Single leaves (Camelina) or leaf discs (*N. benthamiana*) (~20 mg) were taken from the analysed plants and sealed in 1.5 mL GC glass vials using sterilized oven plastic instead of rubber septa. Samples were incubated for 30 min and then the plastic cover was pierced with a Solid Phase Microextraction (SPME) Fiber SUPELCO™ Portable Field Sampler containing a PDMS fibre. The fibre was exposed to the headspace for 30 min and retracted into the SPME sampler afterwards. Samplers containing plant headspace volatiles were placed overnight at 4°C to be manually injected into the GC device. Volatiles were analysed using an Agilent 8890 GC System coupled with an Agilent 5977B mass selective detector. The GC system was equipped with a polar column (HP‐INNOWax, 30 m x 0.25 mm, 0.25 μm film thickness) and was operated using helium as carrier gas (1 mL min^−1^ flow). The method started with the oven temperature set at 50°C for 3 min, then a temperature ramp of 10°C min^−1^ until reaching 230°C and holding it for 1 min.

### 2.8 Compound detection

Detection of pathway intermediates and end‐products was performed by comparing their mass spectra with those from the National Institute of Standards and Technology (NIST17) database library and literature reported profiles for NOL. For NON, apart from NIST17, standard compound was used as reference. NON was synthesized and kindly provided by Adrian Scaffidi and Gavin Flematti (University of Western Australia) (Bol et al., 2022). Software used for total iron current (TIC) and selected ion monitoring analysis was Enhanced ChemStation MSD F.01.03.2357 (Agilent Technologies, Inc). Ion Chromatograms Extraction was performed using the characteristic ions from each standard compound for its identification from the TIC from the samples.

## 3 RESULTS

### 3.1 2A cloning strategy does not result in detectable NON/NOL production, but intermediate geraniol is produced upon GPP precursor supplementation

**FIGURE 2:**
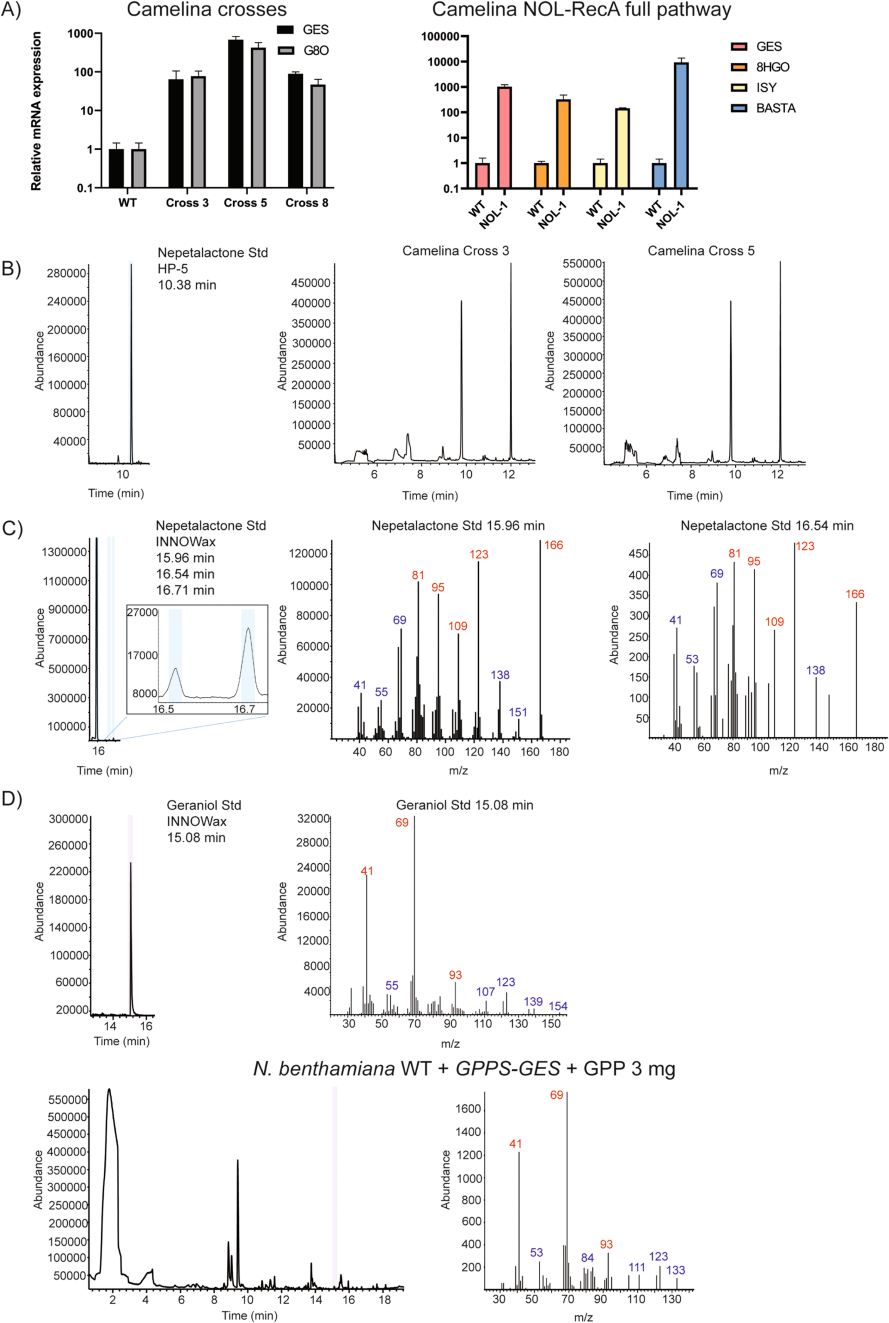
Transcription analysis and volatile profiling of NOL pathway expressing plants. A) Relative mRNA expression of NOL pathway genes in F1 crosses and T1 full‐pathway construct and *C. sativa* (Camelina) backgrounds. WT untransformed plants were used as background reference to normalize mRNA expression. B) Nepetalactone standard (Std) GC/MS retention time, and gas chromatograms for solvent extraction from Camelina. C) Nepetalactone standard (Std) GC/MS retention time and mass spectra after SPME. D) Geraniol standard (Std) GC/MS retention time and mass spectrum for SPME of infiltrated *N. benthamiana* leaf supplemented with GPP precursor. Blue colour indicates detection of nepetalactone at the expected retention time. Purple colour indicates geraniol detection at expected retention time. Ions in font color red represent characteristic fragments for the indicated compound.

In plants, terpenoids are derived from DMAPP and IPP substrates from the MAV and MEP pathway. For NON/NOL production, codon‐optimized *GES, GPPS, G8O, 8‐HGO and ISY* were cloned into two constructs: GES‐GPPS and G8O‐8HGO‐ISY. These multigene constructs were designed using the 2A strategy, in which a single polycistronic mRNA is produced. The 2A sequences between each CDS cause ribosome skipping, resulting in individual proteins being produced. T1 Camelina plants were selected using hygromycin (GES‐GPPS) or Basta (G8O‐8HGO‐ISY) and expression of the introduced genes was confirmed via qRT‐PCR analysis. The plants with the highest transgene expression were selected as parental lines for crossing to obtain all five NOL pathway genes in a single plant. The presence of both constructs was confirmed via qRT‐PCR analysis of one of the genes from each construct (Figure 2 A).

Volatile production was evaluated via solvent extraction and GC/MS analysis. NOL was not detected in any of the Camelina crosses (Figure 2B), nor the intermediate geraniol, which is expected to be detectable via GC/MS as it is a volatile compound. To eliminate the possibility of the analysis method being the cause of no detection, we tested NON reference compound under the same extraction and detection conditions as the leaf samples, and it showed detection and a retention time of 10.38 min (Figure 2B). As a follow‐up, we opted for Solid Phase Microextraction (SPME) fibres to capture the volatiles from the leaf headspace and subsequently inject them into the GC/MS device. NON standard was detected by SPME with its major peak retention time at 15.96 min (Figure 2C). The standard also displayed minor peaks at retention times 16.54 min and 16.71 min, with the mass spectra being very similar to the main peak (Figure 2C). Using SPME, also the standard of the pathway intermediate geraniol was detectable with a retention time of 15.08 min (Figure 2D). However, NON, NOL or geraniol were not detected in any of the analysed transgenic Camelina leaves using SPME analysis of genetically modified plants (data not shown).

To get more insight into the limitations of our strategy, we performed an assay transiently infiltrating *N. benthamiana* with the GES‐GPPS construct. Before collecting leaf samples, the precursor GPP was supplemented to the leaf tissue and incubated for 2 h before exposing it to the SPME fibre. Our analysis showed that, when supplementing with GPP, geraniol was detected from the infiltrated leaf tissue (Figure 2D), indicating that the GES‐GPPS 2A construct was able to produce functional GPPS and GES enzymes *in planta*.

### 3.2 Chloroplast targeting of NOL pathway genes along with an MLPL enzyme results in the production of NON in *N. benthamiana* and *C. sativa*

**FIGURE 3:**
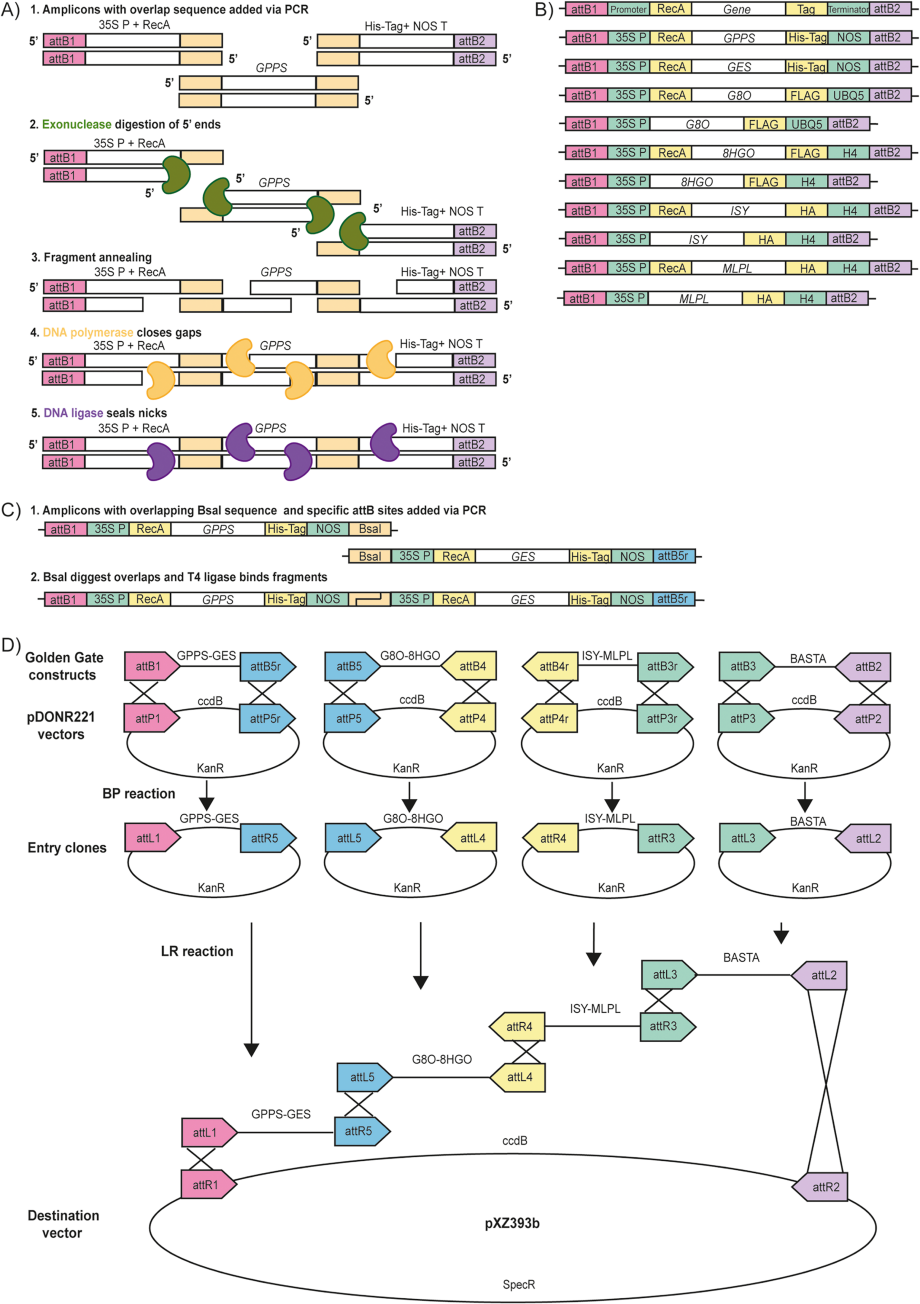
Cloning strategy for full NOL‐pathway constructs + MLPL. A) Gibson Assembly procedure to add 35S promoter, RecA and terminator sequences. B) Products of Gibson Assembly of individual constructs. C) Golden Gate (GG) cloning for creating gene pairs. D) MultiSite Gateway cloning strategy using GG constructs. ccdB: DNA gyrase inhibitor negative selection marker. KanR: kanamycin resistance, SpecR: spectinomycin resistance, 35S P: 35S CaMV promoter, pXZ393b: Destination vector carrying T‐DNA borders for *A. tumefaciens*.

Considering that the 2A cloning strategy could be one of the reasons why NOL production was not achieved, as there could be an issue with the ribosome skipping, we proceeded with an alternative for the genetic engineering of the NOL pathway genes. For this strategy, we cloned each gene with its own CaMV 35S constitutive promoter and different terminators (Figure 3).

As mentioned above, NOL and NON can be derived from DMAPP and IPP, which are produced by the cytosolic MVA pathway and the plastidial MEP pathway. As DMAPP and IPP pools could be restricted in the cytosol due to competition with farnesyl diphosphate synthase (FPPS) (Closa et al., 2010), we evaluated the effect of chloroplast targeting of the enzymes using the RecA chloroplast targeting peptide to access the plastidial DMAPP from the MEP pathway. This strategy was also used by Dudley et al. (2022) when co‐expressing NOL pathway genes transiently by separately infiltrating all the individual steps of the pathway into *N. benthamiana* leaves (Dudley et al., 2022).

Dudley et al. (2022) reported that the best strategy was to target only GPPS and GES to the chloroplast, while the other enzymes were expressed in the cytosol. With this combination, they produced the highest amount of 7‐deoxyloganic acid, which is a product derived from NOL, a step further in the iridoid pathway. Dudley et al. (2022) also reported that incorporating MLPL was important for iridoid synthesis since ISY works in combination with MLPL for proper ring closure of NOL (Lichman et al., 2020; Lichman et al., 2019; Dudley et al., 2022). Therefore, we synthesized and cloned MLPL in the same way as the other NOL pathway genes via Gibson Assembly (Figure 3A). We then created gene pairs via Golden Gate cloning and finally used MultiSite Gateway cloning to create two versions of a 7‐gene construct, each carrying the original five genes of the NOL pathway, MLPL and a Basta resistance gene (Figure 3). Regarding chloroplast targeting, we created a first construct with chloroplast targeting of only GPPS and GES (NOL‐2 RecA), and a second construct having all six NOL‐pathway genes targeted to the chloroplast (NOL‐6 RecA) (Figure 3B).

**FIGURE 4:**
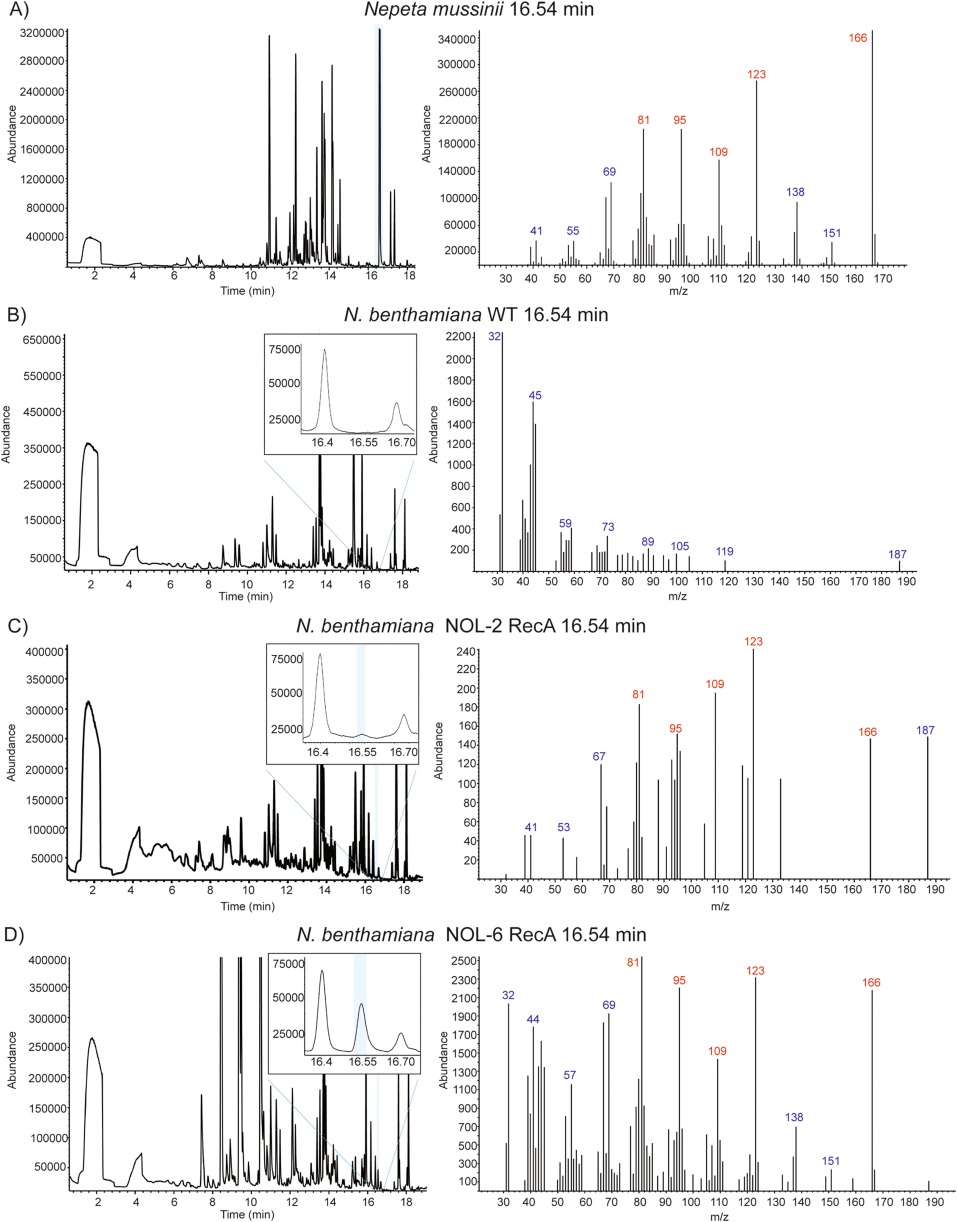
Gas chromatograms and mass spectra of *N. benthamiana* infiltrated with a 7‐gene construct for the NOL pathway and MLPL show matching NON mass spectrum to *N. mussinii*. A) *N. mussinii* positive control, B) Uninfiltrated WT *N. Benthamiana*, C) NOL‐2 RecA: nepetalactol pathway genes + MLPL, targeting only GES and GPPS to the chloroplast, D) NOL‐6 RecA: nepetalactol pathway genes + MLPL, targeting all 6 NOL pathway enzymes to the chloroplast. Blue colour indicates the detection of nepetalactone (NON) at the expected retention time compared to the naturally NON‐producing plant *N. mussinii*. Ions in font color red represent characteristic fragments for the indicated compound.

Both versions of the 7‐gene constructs were transformed stably into Camelina or infiltrated into *N. benthamiana* leaves for transient expression. Relative mRNA expression was confirmed via qRT‐PCR for one gene of each gene pair built via Golden Gate cloning. Overexpression of all tested genes was between 100 and 1000‐fold compared to WT background signal (Figure 2A). NON, but not NOL, was detected using SPME in three transiently transformed *N. benthamiana* samples (Figure 4). For the NON standard, the retention time for the SPME method was expected to be 15.96 min, with additional peaks at 16.54 min and 16.71 min (Figure 2C). In the infiltrated *N. benthamiana* leaves, NON was found for both constructs (NOL‐2 RecA and NOL‐6 RecA) with a matching mass spectrum at 16.54 min containing the expected fragment masses 81, 95, 109, 123 and 166 m/z (Figure 4).

In the case of NOL‐6 RecA, a clear peak eluting at the retention time 16.54 min was observed in the total ion current (TIC), and the mass spectrum matched that from the reference NON compound. As a positive control, we grew *Nepeta mussinii*, a plant with the natural ability to produce iridoid compounds. In *N. mussinii*, we observed a major NON peak at 16.54 min (Figure 4) with a mass spectrum matching the NON standard. In the *N. mussinii* sample, we also did not observe a NON peak at 15.96 min as we had expected from the reference compound (Figure 2C). This could be explained as *N. mussinii* produces mainly *trans‐cis*‐NON, while other species like *Nepeta cataria* produce *cis‐trans*‐NON (Sherden et al., 2018). Indeed, the provided NON standard was the *cis‐* *trans‐*NON as it had been prepared for comparison with *N. cataria* (Bol et al., 2022). For NOL‐2 RecA, only trace amounts of NON were detected. In addition, extracts of non‐infiltrated leaves of *N. benthamiana* were used as negative control. The TIC obtained and the mass spectrum at 16.54 did not match the infiltrated samples nor the *N. mussinii* leaf (Figure 4), further showing that NON production was achieved specifically by introduction of the NOL pathway constructs.

Using the known NON standard concentration (10 ng μL^−1^), we estimated the emitted amount of NON from our *N. benthamiana* leaf samples. We performed an integration analysis and compared the area below the peak against the area below the peak of the standard compound reference. Based on these estimations, NOL‐6 RecA and NOL‐2 RecA emitted approximately 17.4 ng g^−1^ h^−1^ fresh weight (FW) and 4.66 ng g^−1^ h^−1^ FW, respectively.

**FIGURE 5:**
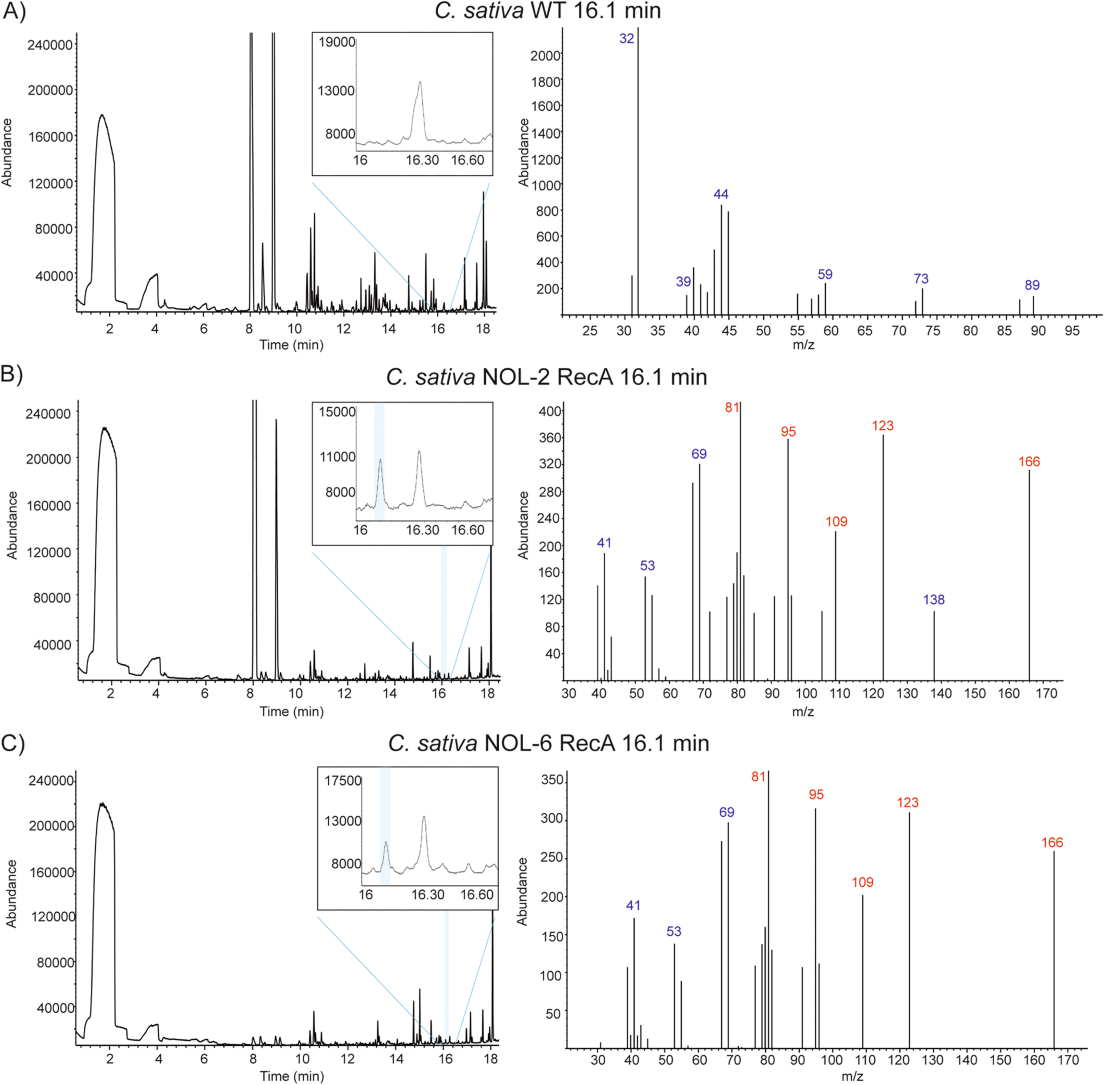
Gas chromatograms and mass spectra of *C. sativa* stably transformed with a 7‐gene construct for the NOL pathway and MLPL show matching NON mass spectra to NON standard. A) Untransformed WT C. sativa, B) NOL‐2 RecA: nepetalactol pathway genes + MLPL, targeting only GES and GPPS to the chloroplast, C) NOL‐6 RecA: nepetalactol pathway genes + MLPL, targeting all 6 NOL pathway enzymes to the chloroplast. Blue colour indicates the detection of mass spectrum matching nepetalactone (NON). Ions in font color red represent characteristic fragments for the indicated compound.

As for Camelina stable transformants, dozens of plants survived antibiotic selection and mRNA expression was confirmed for at least one gene of the gene pairs formed by Golden Gate cloning (Figure 2A). As for volatile detection, two individuals carrying the NOL‐2 RecA construct or NOL‐6 RecA construct, respectively, showed a component with mass spectrum matching that of the NON standard and NON detected from *N. mussinii* (Figure 5), but eluting at different retention time (16.1 min). This might imply the production of another NON isomer. In Camelina WT, there was no detection of NON at any of these mentioned retention times. Quantification analysis showed that the NOL‐2 RecA plant emitted approximately 1.62 ng g^−1^ h^−1^ FW of the compound, while NOL‐6 RecA plant emitted 1.54 ng g^−1^ h^−1^ FW, several times lower than what was observed in transiently transformed *N. benthamiana*. It is worth mentioning that these *Camelina* individuals showed deleterious phenotypes, as they had stunted growth and did not produce seeds before senescence.

## 4 DISCUSSION

Plant genetic engineering for pest control has been established for more than 30 years, with the expression of *Bacillus thuringiensis* (Bt) toxins being the most commercialized strategy to obtain insect‐resistant crops (Gatehouse et al., 2011). However, Bt toxins are not effective against hemipteran pests such as aphids (Chougule and Bonning, 2012). Therefore, other strategies are required for aphid control. Among the most studied forms of aphid control is the use of plant lectins, which have specificity for glycoconjugates in non‐plant organisms (Vandenborre et al., 2011). Several lectin families have shown toxicity to insects by, after ingestion via the plant tissue, coming in contact with carbohydrate structures present in the midgut of insects. This can then cause developmental stunting or mortality (Van Damme et al., 2008; Vandenborre et al., 2011). Different lectins have shown effectiveness against several types of aphids when expressed in many different crops. However, they can also be toxic to non‐target organisms, which could be concerning as animals and humans might consume these crops (Yu et al., 2014). For this reason, it is important to develop alternative aphid control strategies, such as the one described in this study.

In this work, the aphid semiochemical NON was successfully produced in genetically modified plants, both by transient expression in *N. benthamiana* and stable transformation of Camelina. We designed a strategy to build a transferable pathway for NON production into plant systems, building on previous studies of transient expression of this pathway. Here, we advanced the iridoid production field by managing to produce NON without externally supplementing the plants with precursors and by expressing the genes from a single plasmid vector (Dudley et al., 2022; Miettinen et al., 2014). To our knowledge, this is also the first report of engineered NON production in plants that do not natively produce NON, solely using the plant's endogenous metabolite precursor pools.

In the first part of our study, we managed to obtain plants carrying the necessary metabolic genes to produce NOL by using a 2A peptide cloning strategy together with a crossing step. This method, however, did not generate plants that produced the final product NOL, or even the geraniol intermediate. We were thus concerned about the efficiency of the 2A peptide system but, according to literature, this system has proven to be highly efficient in multiple studies and using different variants of these peptides (Liu et al., 2017; Ren et al., 2023). The fact that we observed some geraniol being produced in transiently transformed *N. benthamiana* when externally providing GPP substrate suggests that at least the two‐gene 2A construct produces functional GPPS and GES.

A major limitation may therefore be the amount of substrate available for these enzymes *in vivo*. The introduced enzymes may compete for substrates with other secondary metabolism enzymes such as FPPS in the cytosol while, as stated before, there is an IPP and DMAPP pool also available in the plastids. Dudley et al. (2022) showed that chloroplast targeting of GES and GPPS enzymes was the highest‐yielding combination to boost the production of 7‐DLA, as these enzymes would access the less restricted DMAPP and IPP pool in the plastids. Considering all these reasons, we opted for a cloning strategy with each gene having its own constitutive promoter, including chloroplast targeting peptide, and adding MLPL as a sixth step in the NOL pathway. We produced two vectors carrying all necessary pathway genes (to avoid further crossing), with one version with only GES and GPPS targeted to the chloroplast (NOL‐2 RecA), and one version with all the NOL pathway enzymes targeted to the chloroplast (NOL‐6 RecA).

This strategy resulted in promising outcomes since *N. benthamiana* leaves infiltrated with these constructs showed the presence of NON with a mass spectrum identical to the reference chemical compound and to NON previously identified in *N. mussinii* (Sherden et al., 2018). The retention time matched the NON from *N. mussinii*, but not the major peak in the analysis of the available reference compound, which was *cis‐trans*‐NON as produced by *N. cataria*. This might be due to isomerism, with different plant species preferentially producing a different enantiomer. Nonetheless, the standard compound also showed a minor peak at 16.54 min, which coincided with our infiltrated *N. benthamiana* leaves (Figures 2C and 4) and the major *N. mussinii* NON peak. As *N. mussinii* mainly produces *trans‐cis*‐NON (Sherden et al., 2018), it thus seems that *N. benthamiana* produces this isoform. The observation that the specific enantiomer of produced NON was possibly different in *N. benthamiana* compared to Camelina highlights the value of developing different plant production platforms for obtaining specific isoforms.

In contrast to Dudley et al. (2022), in our study, targeting all enzymes to the chloroplast yielded a clear peak and a matching mass spectrum of NON, while only targeting GES and GPPS chloroplast‐targeted showed only trace amounts. This could be explained by other factors such as measuring different metabolites (7‐DLA vs. NON) or individual growth conditions of the plants, etc. Further research is thus needed to fully understand the effects of differential enzyme targeting on terpenoid yield. Another strategy to improve production could be reducing the amount of precursor that is diverted into competing metabolic pathways. This could potentially be achieved by transformation of the NOL pathway into mutant lines with reduced FPPS expression, which may reduce competition for the IPP and DMAPP substrates (Closa et al., 2010; Keim et al., 2012).

NON production was also achieved in stably transformed *Camelina*, which normally does not produce NON in detectable quantities. The amounts were, however, lower than in the transiently transformed *N. benthamiana* in this study. Transient expression has previously been observed to potentially be higher than stable expression (Wroblewski et al., 2005). One reason could be that transient expression may provide the plant with a higher number of gene copies to translate the enzymes from. As the production of the new compounds may have negative effects on the host plant, there is possibly a lower chance that the plant can successfully inhibit the production or activity of these enzymes post‐transcriptionally in a recently (transiently) transformed tissue, as compared to plants that have adapted their metabolism during their entire life, even across generations. Additionally, co‐infiltration with the P19 suppressor protein may also improve expression levels in the transiently transformed lines as all genes are under CaMV 35S promoter. There have been studies suggesting that this might be a relevant discrepancy between stable and transient expression, also showing that stable transformation may often cause unexpected negative effects for the plant host, which might affect plant development (Stepanenko and Heng, 2017). For example, the T‐DNA insertion could cause a loss of function of a gene, transgene promoters may affect the expression of the surrounding genes and insertion could even cause chromosomal rearrangements (Filipecki and Malepszy, 2006; Forsbach et al., 2003).

Indeed, the two stably transformed Camelina individuals in which we observed NON production were stunted and failed to produce seeds. Approaches such as RNA‐seq, proteomics and metabolomics would be interesting to better understand the negative effects of the transgene expression on plant physiology. This could provide valuable insights into producing new compounds in a way more compatible with plant metabolism.

For future work, there are different improvements that could be made to the genetic engineering presented in this study related to stable transformation. As this is a single vector multi‐gene system, different promoters could be used for each gene to prevent gene silencing throughout generations (Forestier et al., 2021). Another option is the use of inducible promoters (e.g. insect damage‐inducible promoters), which might alleviate the metabolic toll for the plant, only producing the defence compounds when needed (Mishra et al., 2022). Another interesting approach could be the targeted production of NOL and NON in trichomes since these structures are specialized in producing terpenoid compounds and also effectively release them for insect defence (Huchelmann et al., 2017). Similarly, trichome engineering might prevent phytotoxic effects as this structure would serve as storage and would prevent the metabolites from affecting the rest of the plant (Schilmiller et al., 2008).

Although a positive outcome, NON production was somewhat unexpected, as NOL is the direct product of ISY. The conversion from NOL to NON could be performed by an oxidase present already in the plant or due to autoxidation. This has been observed in similar compounds, such as the autoxidation of verbenol to verbenone upon accumulation (Leufvén et al., 1984; Bhattacharyya et al., 1960). Regardless, NON is also a relevant pheromone for aphid control (Lilley and Hardie, 1996) and its production could be further improved in plant systems. There are alternative strategies to try if higher levels of NON/NOL production is possible. For example, ISY could be replaced with nepetalactol‐related short‐chain reductases (NEPS), which are capable of producing specific enantiomers of nepetalactol (Hernández Lozada et al., 2022). At the same time as this project was ongoing, Liu et al. (2024) reported an alternative approach to produce NOL based on the idea that some of the final biosynthesis steps are reversible and intermediates could be metabolized into irreversible unwanted products. To avoid leakage by reverting intermediates, they fused 8HGO and ISY into a single protein, so that the pathway is no longer reversible and nepetalactol accumulated in higher amounts in *Gardenia jasminoides* (Liu et al., 2024). Nonetheless, NOL was never observed in transformed plants nor in positive control *N. mussinii* plants, so conversion of NOL to NON possibly occurs at a very high rate, likely preventing NOL detection *in vivo*. As it seems to be an almost complete conversion, variability and reproducibility of NOL to NON conversion, which could negatively affect the consistent usability of this type of approach, is unlikely.

In this study, we have evaluated the possibility of using plants as biofactories to produce aphid sex pheromones. This would provide new possibilities for aphid control since currently NOL and NON are compounds not abundantly available on the market, and the few alternatives with chemically synthesized compounds are costly. The first possibility for this plant expression system is to optimize NON and/or NOL production to use these plants as biofactories for later purification for use in man‐made baits for manipulation of the behaviour of the pests or their enemies.

Another interesting alternative for plants capable of producing these sex pheromones is their use as natural dispensers. These pheromone‐releasing plants could interfere with aphid mating behaviour since females would be harder to find with the air permeated with plant‐produced pheromones. A third approach is that these plants would allow the recruitment of natural aphid predators, as it has been shown that they are also attracted to sex pheromones to locate their prey (Glinwood et al., 1998; Boo et al., 1998). As this is a proof‐of‐concept study, further work is needed to determine which strategy may be most efficient; for instance, by performing insect choice assays with plants capable of producing aphid sex pheromones, testing the behaviours of both aphids and their predators in both laboratory and field conditions. An alternative test would be the use of asexual aphids, as previous studies showed NON and NOL to have repellent activity towards them (Lu et al., 2024). Interestingly, other insect behaviour studies using our plants might be insightful, as nepeta oil (NON as main component) has shown to be an effective repellent against flies, cockroaches and mosquitos (Sparks et al., 2017; Schultz et al., 2004; Gkinis et al., 2014).

## AUTHOR CONTRIBUTIONS

O.V.A, A.O.C, and A.V.M conceived the project. O.V.A., A.O.C., A.V.M, M.F., and C.L. planned the experiments. A.O.C, J.S., O.M, B.D., H.W. performed the experiments. O.V.A, A.O.C and J.S. analysed the data. A.O.C and O.V.A wrote the manuscript with input from the coauthors.

## FUNDING INFORMATION

This project was supported by NovoNordisk Fonden (NNF18OC0034822, NNF21OC0071628, NNF23OC0084896) and Jörgen Lindström's Foundation. AVM was supported by a Sven and Lily Lawski Foundation postdoctoral stipend (N2017‐0079). OVA was supported by Vetenskaprådet (2017–03854, 2021–04358). CL was supported by Svenska Forskningsrådet Formas (2021–00933).

## Supporting information


**Figure S1.** NOL pathway 2A cloning strategy. A) Synthesized constructs flanked with attB1/2 sites necessary for Gateway cloning. Each gene was codon optimized for A. thaliana. B) Gateway cloning strategy for NOL pathway. KanR: Kanamycin resistance, ccdB: Toxic protein ccdB, SpecR: Spectinomycin resistance, pH2GW7: Destination vector carrying T‐insertion sites for A. tumefaciens and hygromycin resistance for positive selection in plant organisms, pB2GW7: Destination vector carrying T‐insertion sites for A. tumefaciens and Basta resistance for positive selection in plant organisms 2‐gene: GES‐GPPS, 3‐gene: G8O‐8HGO‐ISY. T2A, E2A and P2A = 2A sequences.

**Table S1.** 2A cloning & RT‐qPCR primers used in this project.
**Table S2.** Gibson Assembly primers used in this project.
**Table S3.** Golden Gate cloning primers used in this project.
**Table S4.** MultiSite Gateway primers used in this project.

## Data Availability

The data that support the findings of this study are available from the corresponding author upon reasonable request.
