# Supplementary material for: Plants as biofactories for production of the aphid sex pheromone nepetalactone
Source: Physiol Plant. 2025 Feb 16;177(1):e70110. doi: 10.1111/ppl.70110 (PMC11830648; doi:10.1111/ppl.70110)
Supplement: Supplementary file 1 — Figure S1. NOL pathway 2A cloning strategy. A) Synthesized constructs flanked with attB1/2 sites necessary for Gateway cloning. Each gene was codon optimized for A. thaliana. B) Gateway cloning strategy for NOL pathway. KanR: Kanamycin resistance, ccdB: Toxic protein ccdB, SpecR: Spectinomycin resistance, pH2GW7: Destination vector carrying T‐insertion sites for A. tumefaciens and hygromycin resistance for positive selection in plant organisms, pB2GW7: Destination vector carrying T‐insertion sites for A. tumefaciens and Basta resistance for positive selection in plant organisms 2‐gene: GES‐GPPS, 3‐gene: G8O‐8HGO‐ISY. T2A, E2A and P2A = 2A sequences. [file PPL-177-e70110-s002.pdf]

Plants as biofactories for production of the aphid sex pheromone nepetalactone

Abraham Ontiveros-Cisneros[1], Jule Salfeld[1,2], Bao-Jian Ding[1], Hong-Lei Wang[1], Oliver Moss[1,3], Magne Friberg[1], Alex Van Moerkercke[1], Christer Löfstedt[1] & Olivier Van Aken[1*]

[1] Department of Biology, Lund University, Lund, Sweden

[2] Current address: Faculty of Biology, University of Freiburg, Freiburg, Germany

[3] Current address: Plant Breeding Department, Swedish University of Agricultural Sciences, Alnarp, Sweden

*Corresponding author Olivier Van Aken

Molecular Cell Biology Unit

Department of Biology

Lund University

Sölvegatan 35

Lund 223 62 – Lund, Sweden

Tel: +46 76 210 14 03

E-mail: olivier.van_aken@biol.lu.se

Supporting information

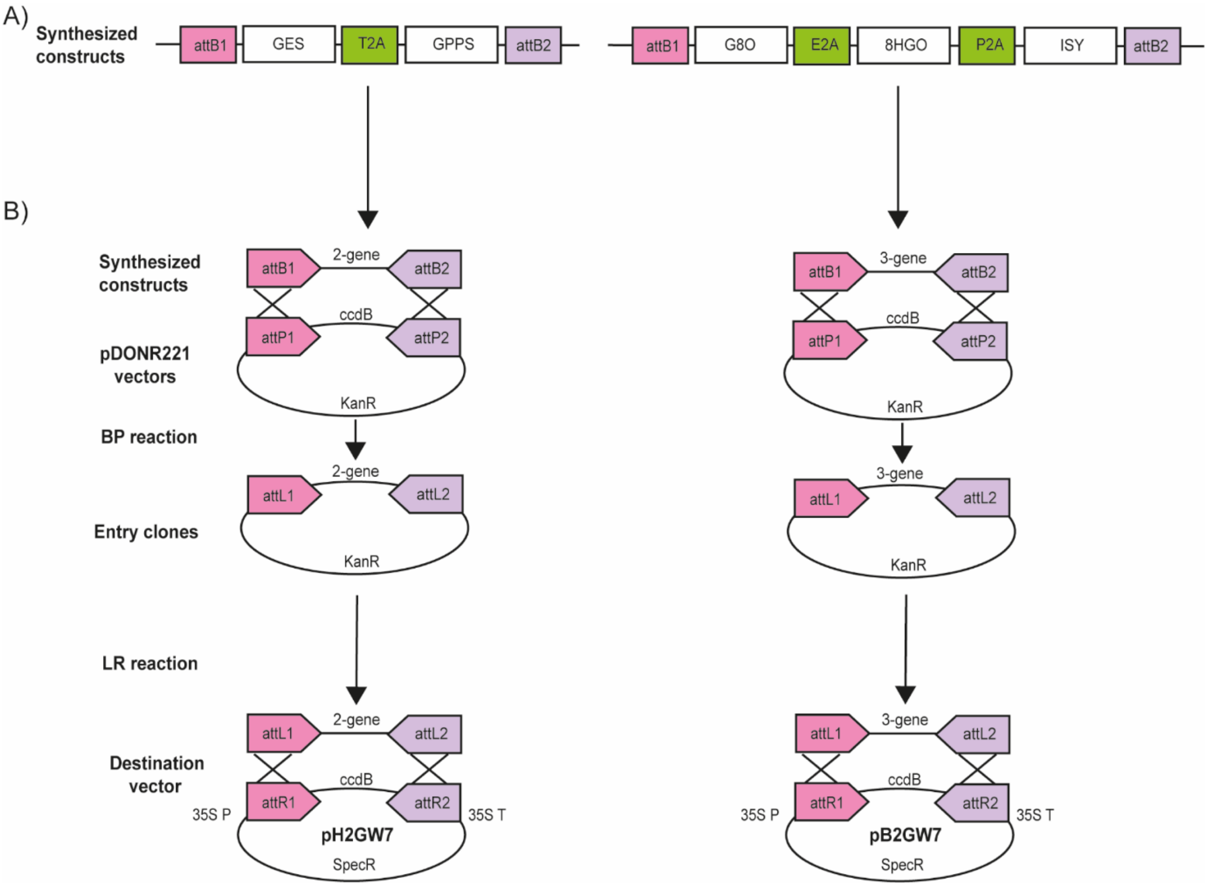

Figure S1. NOL pathway 2A cloning strategy. A) Synthesized constructs flanked with attB1/2 sites necessary for Gateway cloning. Each gene was codon optimized for *A. thaliana*. **B)** Gateway cloning strategy for NOL pathway. KanR: Kanamycin resistance, ccdB: Toxic protein ccdB, SpecR: Spectinomycin resistance, pH2GW7: Destination vector carrying T-insertion sites for *A. tumefaciens* and Hygromycin resistance for positive selection in plant organisms, pB2GW7: Destination vector carrying T-insertion sites for *A. tumefaciens* and Basta resistance for positive selection in plant organisms 2-gene: *GES-GPPS*, 3-gene: *G8O-8HGO-ISY*. T2A, E2A and P2A= 2A sequences.
